# Supplementary material for: Analysis of PPARγ Signaling Activity in Psoriasis
Source: Int J Mol Sci. 2021 Aug 10;22(16):8603. doi: 10.3390/ijms22168603 (PMC8395241; doi:10.3390/ijms22168603)
Supplement: Supplementary file 1 [file ijms-22-08603-s001.zip › Supplemental materials_Analysis of PPARg signaling activity in psoriasis/Pathway models/Models images and html files/PPARG inhibits phenotypes assotiated with psoriasis/234629.html]

cytokine response


# Cell Process cytokine response

|  |  |
| --- | --- |
| URN | urn:agi-cellproc:cytokine%20response |
| Total Entities | 0 |
| Connectivity | 1663 |
| Name | cytokine response |

---

|  |  |
| --- | --- |
| Pathway | SOCS in Negative Feedback Regulation in Myocarditis |
|  | PPARG inhibits phenotypes assotiated with psoriasis |

---

|  |  |
| --- | --- |
| MedScan ID | 4293871 |

---

|  |  |
| --- | --- |
| Alias | cytokine response |
|  | cytokine responses |

---
